# Flavonoids as tyrosinase inhibitors in *in silico* and *in vitro* models: basic framework of SAR using a statistical modelling approach

**DOI:** 10.1080/14756366.2021.2014832

**Published:** 2021-12-20

**Authors:** Katarzyna Jakimiuk, Suat Sari, Robert Milewski, Claudiu T. Supuran, Didem Şöhretoğlu, Michał Tomczyk

**Affiliations:** a Department of Pharmacognosy, Faculty of Pharmacy with the Division of Laboratory Medicine, Medical University of Białystok, Białystok, Poland; b Department of Pharmaceutical Chemistry, Faculty of Pharmacy, Hacettepe University, Ankara, Turkey; c Department of Statistics and Medical Informatics, Faculty of Health Science, Medical University of Białystok, Białystok, Poland; d Neurofarba Department, Universita degli Studi di Firenze, Florence, Italy; e Department of Pharmacognosy, Faculty of Pharmacy, Hacettepe University, Ankara, Turkey

**Keywords:** Flavonoid, molecular docking, tyrosinase, structure-activity relationship, statistical analysis

## Abstract

Flavonoids are widely distributed in plants and constitute the most common polyphenolic phytoconstituents in the human diet. In this study, the *in vitro* inhibitory activity of 44 different flavonoids (**1–44**) against mushroom tyrosinase was studied, and an *in silico* study and type of inhibition for the most active compounds were evaluated too. Tyrosinase inhibitors block melanogenesis and take part in melanin production or distribution leading to pigmentation diseases. The *in vitro* study showed that quercetin was a competitive inhibitor (IC_50_=44.38 ± 0.13 µM) and achieved higher antityrosinase activity than the control inhibitor kojic acid. The *in silico* results highlight the importance of the flavonoid core with a hydroxyl at C7 as a strong contributor of interference with tyrosinase activity. According to the developed statistical model, the activity of molecules depends on hydroxylation at C3 and methylation at C8, C7, and C3 in the benzo-*γ*-pyrane ring of the flavonoids.

## 1. Introduction

Flavonoids are secondary plant metabolites that can be chemically divided into groups based on their substitutions. Flavonoid moieties can be modified by glycosylation, hydrogenation, hydroxylation, acetylation and methylation, as well as by malonylation and sulfatation. The chemical and biological potentials of flavonoids and their derivatives are connected with the position of diverse substitutions on the molecule and the saturation of double bonds in the structure^1^. Thus, structure-activity relationship (SAR) studies can help predict the biological activities of compounds with related structures and help avoid off-target outcomes due to their toxicity and side effects. The substantial contribution of a treatment is based on selecting the right medicine for a given target. Available theoretical tools (e.g., molecular docking studies) can help predict potential inhibitory activity against enzymes, including tyrosinase, and virtual screening can be used to select compounds that target tyrosinase and to determine expected targets for well-known and newly discovered flavonoids^2^^,^^3^. Tyrosinase is common in mammals, fungi, bacteria, and plants, and it plays a critical role in melanin biosynthesis. Tyrosinase consists of two identical H subunits as a catalytic component and two identical L subunits. The H subunit includes four helices that contain the catalytic binuclear copper-binding site. Each Cu^2+^ cofactor forms coordination bonds with three histidine residues (His61, His85, His94, and His259, His263, His296, respectively) located at the centre of two antiparallel α helix pairs^4^^,^^5^. His85 is covalently bound to Cys83 through a thioether bond, and these two residues are connected to each other via a threonine residue that forms a triad motif that is conserved among tyrosinases and is considered essential for catalytic activity^6^^,^^7^. The histidine ligands of the copper ions are stabilised via interactions with nearby residues such as Phe90 and Phe292 for catalytic activity; thus, interactions with the copper ions as well as their ligands and the nearby residues are required for effective inhibition^8^^,^^9^. In humans, abnormal melanin production or distribution leads to pigmentation diseases, such as overtanning, freckles, age spots, and melasma. Disorderly melanin production plays a key role in melanotic melanoma, and inhibiting tyrosinase activity may reduce melanin content and be a useful process in skin-whitening compounds^10^^,^^11^.

In this study, we investigated the tyrosinase inhibitory activity of 44 flavonoids, (7-methoxynaringenin (**1**), 7,4′-dimethoxynaringenin (**2**), butin (**3**), isookanin (**4**), hesperetin (**5**), hesperetin 7-*O*-rutinoside (hesperidin) (**6**), 5-hydroxyflavone (**7**), 5-hydroxy-2′-methoxyflavone (**8**), 5-hydroxy-2′,6′-dimethoxyflavone (**9**), zapotin (**10**), chrysin (**11**), apigenin (**12**), apigenin 7-*O*-glucoside (cosmosiin) (**13**), apigenin 8-*C*-glucoside (vitexin) (**14**), apigenin 8-*C*-glucosyl-2"-*O*-glucoside (**15**), acacetin (**16**), luteolin (**17**), luteolin 7-*O*-glucoside (cynaroside) (**18**), luteolin 7-*O*-sambubioside (**19**), luteolin-6-*C*-glucoside (isoorientin) (**20**), chrysoeriol (**21**), 5,7,3′-trihydroxy-4′-methoxyflavone-8-*C*-xylopyranoside-2"-*O*-glucoside (scleranthoside A) (**22**), 5,7,3′-trihydroxy-4′-acetoxyflavone-8-*C*- xylopyranoside-2"-*O*-glucoside (scleranthoside B) (**23**), 5,7-dihydroxy-3′-methoxy-4′-acetoxyflavone-8-*C*-xylopyranoside-2"-*O*-glucoside (**24**), 5,7-dihydroxy-3′-methoxy-4′-acetoxyflavone-8-*C*-xylopyranoside-2"-*O*-(4′"-acetoxy)-glucoside (scleranthoside D) (**25**), kaempferol (**26**), 8-methoxykaempferol (**27**), kumatakenin (**28**), kaempferol 3-*O*-glucoside (astragalin) (**29**), kaempferol 3-*O*-glucuronide (**30**), kaempferol 3-*O*-galactoside (hyperoside) (**31**), kaempferol 3-*O*-(6″-*O*-*trans*-*p*-coumaroyl)-glucoside (tiliroside) (**32**), ermanin (**33**), icariin (**34**), quercetin (**35**), quercetin 3-*O*-glucuronide (miquelianin) (**36**), quercetin 3-*O*-rutinoside (rutin) (**37**), quercetin 3-*O*-rutinoside-7-*O*-glucoside (**38**), 7-*O*-methoxyquercetin (rhamnetin) (**39**), 7-*O*-methoxyquercetin (isorhamnetin) (**40**), robinetin (**41**), myricetin (**42**), daidzein (**43**), and genistein (**44**), to find potentially active compounds. First, tyrosinase inhibition and enzyme kinetics were tested in an *in vitro* model to determine the types of inhibition for the compounds with the highest activity. Second, in an *in silico* model, docking studies were performed to describe SARs, which were also assessed using a statistical model. Thus, a combination of bioinformatics simulation and biological *in vitro* studies describe the functional mechanisms of the tested compounds.

## 2. Materials and methods

### 2.1. Chemical and reagents

Kojic acid (CAS number: 501-30-4), 3,4-dihydroxy-L-phenylalanine (L-DOPA) (CAS number: 59-92-7; purity ≥98%), and tyrosinase from mushrooms (CAS number: 9002-10-2) were purchased from Sigma–Aldrich Chemical Co. (St. Louis, MO, USA). Compounds **5** (CAS number: 69097-99-0), **11** (CAS number: 480-40-0), **12** (CAS number: 520-36-5), **14** (CAS number: 3681-93-4), **20** (CAS number: 4261-42-1), **21** (CAS number: 491-71-4), **26** (CAS number: 520-18-3), **35** (CAS number: 6151-25-3), **39** (CAS number: 90-19-7), **40** (CAS number: 480-19-3), **42** (CAS number: 529-44-2) and **44** (CAS number: 446-72-0) were obtained from Carl Roth (Karlsruhe, Germany), **16** (CAS number: 480-44-4), **34** (CAS number: 489-32-7), and **43** (CAS number: 552-66-9) were obtained from Cayman Chemical (Ann Arbour, MI, USA). Flavonoids **1**, **2**, **28**, **33**^12^, **13**^13^, **15**, **31**, **38**^14^, **17**^15^, **18**, **19**^16^, **7**, **8**, **9**^17^**, 22**, **23**, **24**, **25**^18^, **27**, **32**^19^, **29**, and **37**^20^ were isolated and identified in the Department of Pharmacognosy at the Medical University of Białystok. Compounds **3** (CAS number: 492-14-8), **4** (CAS number: 1036-49-3), and **10** (CAS number: 14813-19-5) were purchased from Biosynth Carbosynth (London, UK), **6** (CAS number: 520-26-3) and **41** (CAS number: 490-31-3) were purchased from Fluka Analysis (St. Louis, MO, USA), and **30** (CAS number: 22688-78-4) and **36** (CAS number: 22688-79-5) were obtained from Extrasynthese (Genay, France). All isolated compounds were >95% pure as measured by HPLC. Bioassay was performed on BioTek Instruments microplate spectrophotometer EPOCH 2 (Oxfordshire, UK).

### 2.2. Tyrosinase inhibition assay

The inhibitory effect on mushroom tyrosinase (TYase) was evaluated using a method reported in the literature with some modifications^21^. This assay was performed in PBS (100 mM, pH = 6.8 in 25 °C). Test samples **1**–**44** (80 µL) were preincubated with TYase solution (40 µL; 250 U/mL) at 25 °C for 10 min. Then, L-DOPA (80 µL; 0.19 mg/mL) was added, and after an additional 10 min at 25 °C, the absorbance was measured at 492 nm. A blank analysis was performed using PBS instead of a sample, and the positive control was conducted with kojic acid. The inhibitory effect was calculated as follows:

TYaseInh (%) = [(B−C)/C] x 100

where B and C are the absorbances of the blank and the compounds, respectively. The compound concentration that inhibits 50% of tyrosinase activity (IC_50_) was calculated. All tests were performed in triplicate.

### 2.3. Tyrosinase inhibition kinetic analysis

Based on IC_50_ values, the seven most active compounds (**4**, **17**, **26**, **32**, **35**, **41**) were selected for kinetic analysis. The enzyme reaction kinetics of these compounds were measured by constructing Lineweaver-Burk plots of inverse velocities (1/*V*), contrary to the inverse of substrate concentration (1/*S*)^22^^,^^23^. Preincubations and measurement times were performed using the same protocol as described above. The enzyme concentration (250 U/mL) was kept constant in the presence of substrate solutions (L-DOPA) between 0.25 and 2 mM in all kinetic studies. The inhibitor concentrations for all test compounds were 0, 25, and 50 µM. The inhibitory types and inhibitory constant (*K_i_*) values were described by 1/*V* versus inhibitor concentration plots (Dixon plot).

### 2.4. Molecular docking

The selected flavonoids were sketched in ACD/ChemSketch to build and generate their mol topology format. The compounds were modelled using LigPrep (2021–22, Schrödinger LLC, New York, NY) and MacroModel (2021–22, Schrödinger LLC, New York, NY) according to the OPLS4 forcefield parameters^24^ and conjugate gradients method. The original configurations of the ligands were conserved, and their ionisation states at pH 7 were modelled. The mushroom tyrosinase structure (PDB ID: 2Y9X^5^; resolution: 2.78 Å) was downloaded from the RCSB protein data bank (www.rcsb.org)^25^ and prepared for docking using the Protein Preparation Wizard of Maestro (2021–22, Schrödinger LLC, New York, NY)^26^. In this process, redundant molecules were removed, H atoms were added, partial charges were assigned, and ionisation, tautomeric states, and H bonds were set. The active site grids were generated using the Receptor Grid Generator panel of Maestro by setting the central coordinates as −0.62, 26.99, and −43.78, and the volume as 27.000 Å^3^. The ligand was docked to the active site using Glide (2021-2, Schrödinger LLC, New York, NY) in additional precision (XP) mode with 50 runs per ligand, and the results were visually evaluated^27^. Prior to docking of the ligands, the co-crystallized ligand in the PDB structure, tropolone, was removed and redocked to the active site, and the obtained binding mode was compared with the co-crystallized conformer by calculating the root-mean-square deviation (RMSD) to confirm the precision of the method. The predicted binding mode for tropolone was similar to that of the original conformer (RMSD: 1.33 Å).

### 2.5. Statistical analysis

Statistical analysis for IC_50_ calculations was performed using nonlinear regression using GraphPad Prism 9 (Trail, GraphPad Software, La Jolla, CA, USA). Data were collected as mean ± SD (*n* = 3), and the significance of differences was analysed using one-way analysis of variance (ANOVA). Statistical analysis of SARs was performed using Stata/IC 13.1 (StataCorp LP, TX 77845, USA).

## 3. Results and discussion

### 3.1. Inhibitory effects of tested compounds on tyrosinase

**Figure 1.**
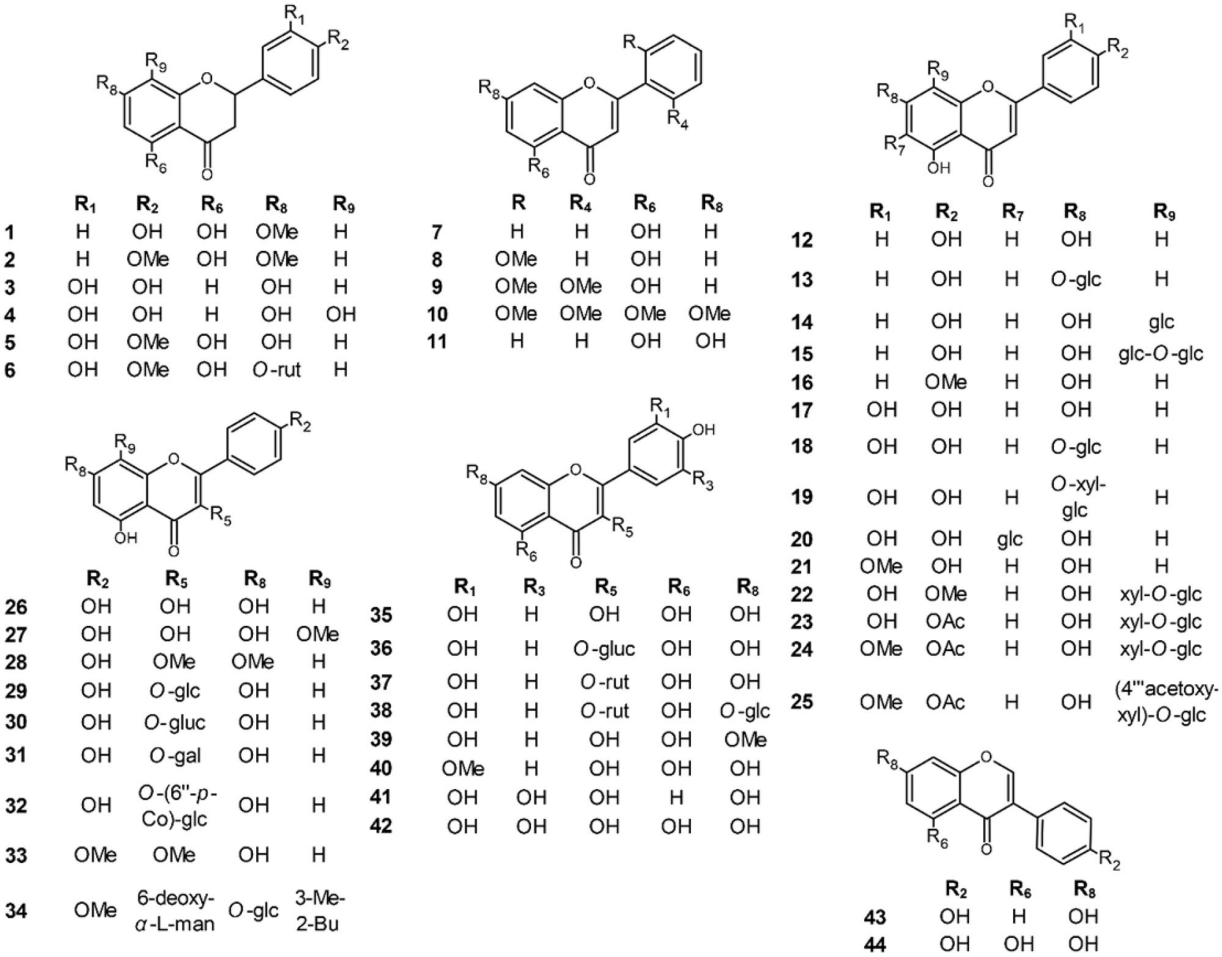
Molecular structures of the tested compounds.

The effects of different concentrations of selected flavonoids (Figure 1) and kojic acid, a well-known inhibitor of L-DOPA oxidation by tyrosinase, were studied. The effects, expressed as IC_50_ values, are shown in Table 1.

**Table 1.** Compounds **1**–**44** measured for anti-tyrosinase activity and their respective IC_50_ values.

No	π C2-C3	B-ring					Benzo-*y*-pyrane ring					IC_50_ (µM)
		C2’	C3’	C4’	C5’	C6’	C3	C5	C6	C7	C8	
		(R)	(R_1_)	**(R_2_)**	**(R_3_)**	**(R_4_)**	(R_5_)	(R_6_)	(R_7_)	(R_8_)	(R_9_)	
**1**	–	H	H	OH	H	H	H	OH	H	OMe	H	441.92 ± 1.91
**2**	–	H	H	OMe	H	H	H	OH	H	OMe	H	>500
**3**	–	H	OH	OH	H	H	H	H	H	OH	H	296.64 ± 2.81
**4**	–	H	OH	OH	H	H	H	H	H	OH	OH	58.43 ± 0.38
**5**	–	H	OH	OMe	H	H	H	OH	H	OH	H	237.49 ± 2.63
**6**	–	H	OH	OMe	H	H	H	OH	H	*O*-rut	H	130.71 ± 1.40
**7**	+	H	H	H	H	H	H	OH	H	H	H	120.45 ± 1.48
**8**	+	OMe	H	H	H	H	H	OH	H	H	H	122.06 ± 1.19
**9**	+	OMe	H	H	H	OMe	H	OH	H	H	H	127.00 ± 1.40
**10**	+	OMe	H	H	H	OMe	H	OMe	H	OMe	H	137.16 ± 1.44
**11**	+	H	H	H	H	H	H	OH	H	OH	H	129.42 ± 1.13
**12**	+	H	H	OH	H	H	H	OH	H	OH	H	160.52 ± 1.44
**13**	+	H	H	OH	H	H	H	OH	H	*O*-glc	H	323.23 ± 1.48
**14**	+	H	H	OH	H	H	H	OH	H	OH	glc	133.07 ± 1.83
**15**	+	H	H	OH	H	H	H	OH	H	OH	glc-*O*-glc	159.26 ± 1.17
**16**	+	H	H	OMe	H	H	H	OH	H	OH	H	207.86 ± 1.35
**17**	+	H	OH	OH	H	H	H	OH	H	OH	H	58.88 ± 0.78
**18**	+	H	OH	OH	H	H	H	OH	H	*O*-glc	H	415.00 ± 2.38
**19**	+	H	OH	OH	H	H	H	OH	H	*O*-xyl-glc	H	159.26 ± 1.17
**20**	+	H	OH	OH	H	H	H	OH	glc	OH	H	132.55 ± 2.32
**21**	+	H	OMe	OH	H	H	H	OH	H	OH	H	>500
**22**	+	H	OH	OMe	H	H	H	OH	H	OH	xyl-*O*-glc	>500
**23**	+	H	OH	OAc	H	H	H	OH	H	OH	xyl-*O*-glc	>500
**24**	+	H	OMe	OAc	H	H	H	OH	H	OH	xyl-*O*-glc	>500
**25**	+	H	OMe	OAc	H	H	H	OH	H	OH	(4′''acetoxy-xyl)-*O*-glc	>500
**26**	+	H	H	OH	H	H	OH	OH	H	OH	H	65.11 ± 1.09
**27**	+	H	H	OH	H	H	OH	OH	H	OH	OMe	290.46 ± 1.19
**28**	+	H	H	OH	H	H	OMe	OH	H	OMe	H	>500
**29**	+	H	H	OH	H	H	O-glc	OH	H	OH	H	481.54 ± 1.73
**30**	+	H	H	OH	H	H	*O-*gluc	OH	H	OH	H	269.97 ± 1.23
**31**	+	H	H	OH	H	H	*O*-gal	OH	H	OH	H	186.91 ± 1.09
**32**	+	H	H	OH	H	H	*O*-(6′'-*p*-Co)-glc	OH	H	OH	H	78.53 ± 0.84
**33**	+	H	H	OMe	H	H	OMe	OH	H	OH	H	231.72 ± 1.17
**34**	+	H	H	OMe	H	H	6-deoxy-*α*-L-man	OH	H	*O*-glc	3-Me-2-Bu	150.01 ± 1.84
**35**	+	H	OH	OH	H	H	OH	OH	H	OH	H	44.38 ± 0.13
**36**	+	H	OH	OH	H	H	*O*-gluc	OH	H	OH	H	220.10 ± 1.14
**37**	+	H	OH	OH	H	H	*O*-rut	OH	H	OH	H	141.67 ± 1.30
**38**	+	H	OH	OH	H	H	*O*-rut	OH	H	*O*-glc	H	147.65 ± 1.57
**39**	+	H	OH	OH	H	H	OH	OH	H	OMe	H	170.71 ± 2.05
**40**	+	H	OMe	OH	H	H	OH	OH	H	OH	H	>500
**41**	+	H	OH	OH	OH	H	OH	H	H	OH	H	63.14 ± 0.85
**42**	+	H	OH	OH	OH	H	OH	OH	H	OH	H	100.33 ± 1.86
**43**	+	H	H	OH	H	H	H	H	H	OH	H	>500
**44**	+	H	H	OH	H	H	H	OH	H	OH	H	>500

Abbreviations: π C2-C3 +/– present or absence of double bond; Me: methyl; Bu: butenyl; Ac: acetyl; glc: glucose; gluc: glucuronide; *p*-Co: *para*-coumaroyl; man: mannose; rut: rutinose; xyl: xylose.

Although the rendered activity is accumulative of an entire molecule, a structure-activity relationship was recognised by examining the effect of different substitutions: the presence or absence of hydroxyl groups or their methylation/acetylation/glycosylation in all carbons of the B-ring, as well as in carbons 3, 5, 6, 7, and 8 of the benzo-*γ*-pyrane ring, on the potential inhibitory activity.

Some flavonoids (**4**, **17**, **35**) exhibited IC_50_ values that were comparable to the positive control, kojic acid (IC_50_=49.48 ± 0.23 µM). Compound **4** (IC_50_=58.43 ± 0.38 µM), tested in this study for the first time, was one of the most effective inhibitors of tyrosinase, along with compound **17** (IC_50_=58.88 ± 0.78 µM) and compound **35** (IC_50_=44.38 ± 0.13 µM). The distinction in position C3 in **35** and **36** (IC_50_=220.10 ± 1.14 µM) or **37** (IC_50_=141.67 ± 1.30 µM) is only a difference in the glycosylation of a hydroxyl group. However, this small difference in their structure causes immense differences in their inhibitory potentials. It is presumed that substitutions at C3 led to steric hindrances that prevent molecules from binding to enzymes. However, the most typical locations for C-glycosyl radicals are the C6 and C8 positions at the A-ring. It seems that aglycone **4 (**IC_50_=58.43 ± 0.38 µM) is a more effective tyrosinase inhibitor than it glycoside: **20** (IC_50_=132.55 ± 2.32 µM). The exact location of the glycosidic residue (carbon 6 or 8) does not affect activity. Also, disaccharides linked with the flavonoid core in C8 (**22**, **23**, **24**, **25**) exhibit weak inhibitory potential with IC_50_ values that are higher than those of the maximum tested concentration. When the antityrosinase activity of methylated **26**, **27**, and **28** molecules with IC_50_ values 65.11 ± 1.09 µM, 290.46 ± 1.19 µM, and >500 µM, respectively, was compared, the presence of methyl groups likely prevents appropriate interactions with the enzyme active site^28^. The same SAR pattern was observed with compounds **4** (without methylation), **1** (one methyl group at C7), and **2** (two methyl groups at C7, C4’), with IC_50_ values of 58.43 ± 0.38 µM, 441.92 ± 1.91 µM, and >500 µM, respectively. The links of the B-ring in the C-ring allow flavone and isoflavone activity to be compared. Isoflavones (3-B-ring) with IC_50_ values >500 µM (**43** and **44**) exhibit no inhibitory potential. Additionally, the confrontation of the activity of **35** (IC_50_=44.38 ± 0.13 µM) with **42** (IC_50_=100.33 ± 1.86 µM) leads to the conclusion that the additional hydroxyl group in C5' reduces phenol inhibitory potency. It was not possible to determine the IC_50_ values of flavonoids **2**, **21**, **22**, **23**, **25**, **28**, **40**, **43** and **44** up to 500 µM, which was the highest tested concentration. Thus, based on the *in vitro* study, molecules containing more unsubstituted hydroxyl groups in C3, C5, and C7, as well as in C3' and C4', generally achieve highly potent activity against tyrosinase, this finding is consistent with previous study^11^. Similar conclusions of SAR were presented using inhibitors of xanthine oxidase and elastase and scavengers of superoxide radicals^29^^,^^30^.

### 3.2. Kinetic analysis of tyrosinase inhibition

**Figure 2.**
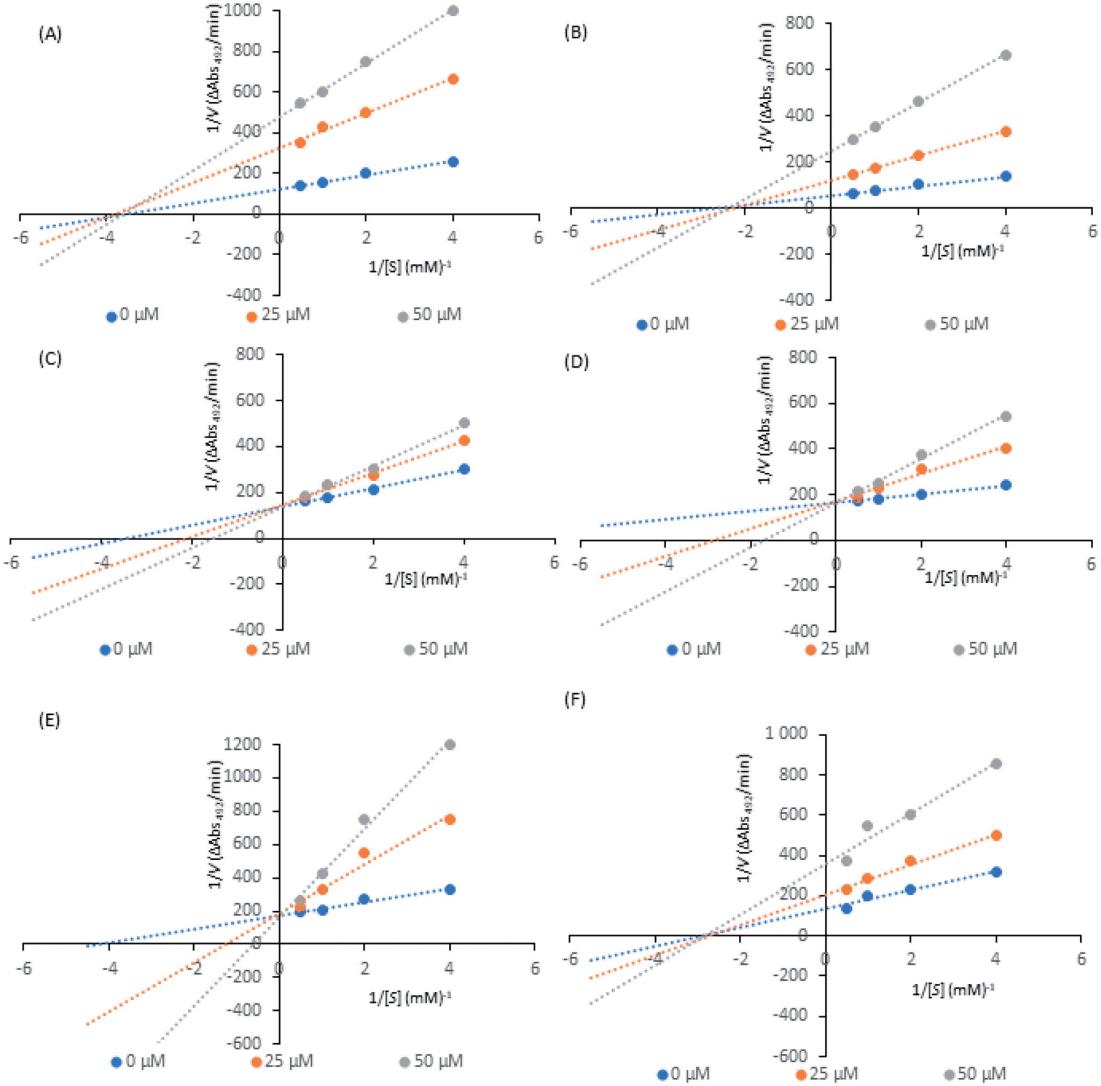
Lineweaver–Burk plots for inhibition of tyrosinase in the presence of compounds: **4** (A), **17** (B), **26** (C), **32** (D), **35** (E), and **41** (F). The concentrations of the compounds were 0.00, 25, and 50 µM. The substrate L-DOPA concentrations were 0.25, 0.50, 1, and 2 mM.

**Figure 3.**
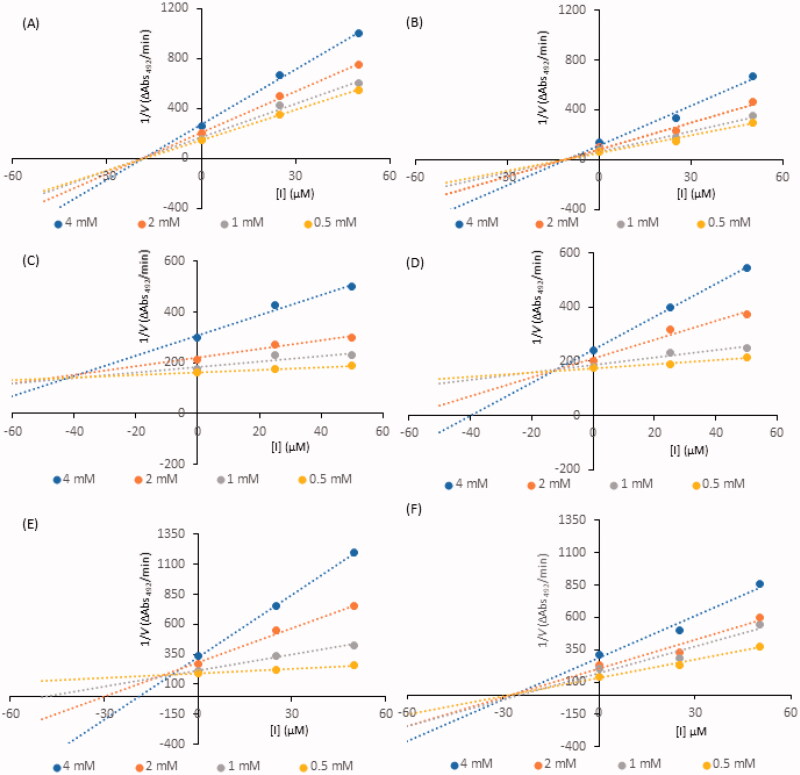
Dixon plots for inhibition of tyrosinase in the presence of compounds: **4** (A), **17** (B), **26** (C), **32** (D), **35** (E), and **41** (F). The concentrations of the compounds were 0.00, 25, and 50 µM. The substrate L-DOPA concentrations were 0.25, 0.50, 1, and 2 mM.

Kinetic analysis of compound-induced inhibition was performed to determine the type of inhibition of the most active constituents (i.e., IC_50_ values below 100 µM). Lineweaver-Burk plots (Figure 2) in double-reciprocal form and Dixon plots (Figure 3) were used to determine the inhibition types and evaluate the dissociation constants for the inhibitors (K_i_) (Table 2). The inhibitory type of isookanin (**4**) and robinetin (**41**) was tested for the first time in this study.

**Table 2.** Kinetic analysis of active compounds on tyrosinase.

Compound	Type of inhibition	K_i_ (µM)^a^
**4**	non-competitive	18.64 ± 0.53
**17**	non-competitive	11.32 ± 0.77
**26**	competitive	40.20 ± 0.46
**32**	competitive	9.83 ± 0.57
**35**	competitive	8.21 ± 0.86
**41**	non-competitive	27.42 ± 0.62

^a^All data are represented as Ki values with standard deviation from triplicate measurements.

As shown in Figure 2(A,B,F), the straight lines intersected at the same point on the x-axis, implying that the *K_m_* value remained constant, while the maximum reaction rate (*V_max_*) decreased with increasing concentrations of tested inhibitors (0, 25, and 50 µM). Thus, isookanin (**4**), luteolin (**17**) and robinetin (**41**) likely caused non-competitive tyrosinase inhibition. Compounds **4**, **17** and **41** produce allosteric regulation, which is a specific type of enzyme inhibition that is characterised by an inhibitor binding to an allosteric site, resulting in decreased enzyme efficacy. The luteolin type of inhibition was consistent with previous studies^31^. The inhibition constant K_i_ was obtained from Dixon plots (Figure 3(A,B,F)) as 18.64 ± 0.53 µM, 11.32 ± 0.77 µM and 27.42 ± 0.62 µM for compounds **4**, **17**, and **41**, respectively.

In contrast, kaempferol (**26**), tiliroside (**32**) and quercetin (**35**) exhibit tyrosinase inhibition that are similar^11^^,^^32^^,^^33^. As shown in Figure 2(C,D,E), all straight lines crossed at the same point on the y-axis, which indicated that *V_max_* remained constant. As the x-axis intercepted increased with increasing inhibitor concentrations, *K_m_* increased because a higher concentration of substrate is required to overcome the inhibitory effects of a competitor. Thus, compounds **26**, **32**, **35** can bind to the active site and prevent binding to the real substrate. Based on Dixon plots (Figure 3(C–E)), *K_i_* for compounds **26**, **32**, **35** were calculated to be 40.20 ± 0.46 µM, 9.83 ± 0.57 µM, and 8.21 ± 0.86 µM, respectively. The inhibition constant for quercetin (**35**) was the lowest, suggesting that the inhibitory effect of this compound is leading among all tested flavonoid structures. This result is consistent with the tyrosinase inhibitory activity test (IC_50_=44.38 ± 0.13 µM).

### 3.3. Molecular docking studies

Active compounds were generally predicted to show high affinity to the active site, as represented by their docking scores (Table 3). Compounds **26** and **41** were particularly noteworthy based on their docking scores, which were based on a set of profitable interactions with the enzyme and its cofactors.

**Table 3.** Docking scores of the active flavonoids.

Compound	Docking score (kcal/mol)
**4**	−6.6
**17**	−7.9
**26**	−9.1
**32**	−8.5
**35**	−7.9
**41**	−10.3

**Figure 4.**
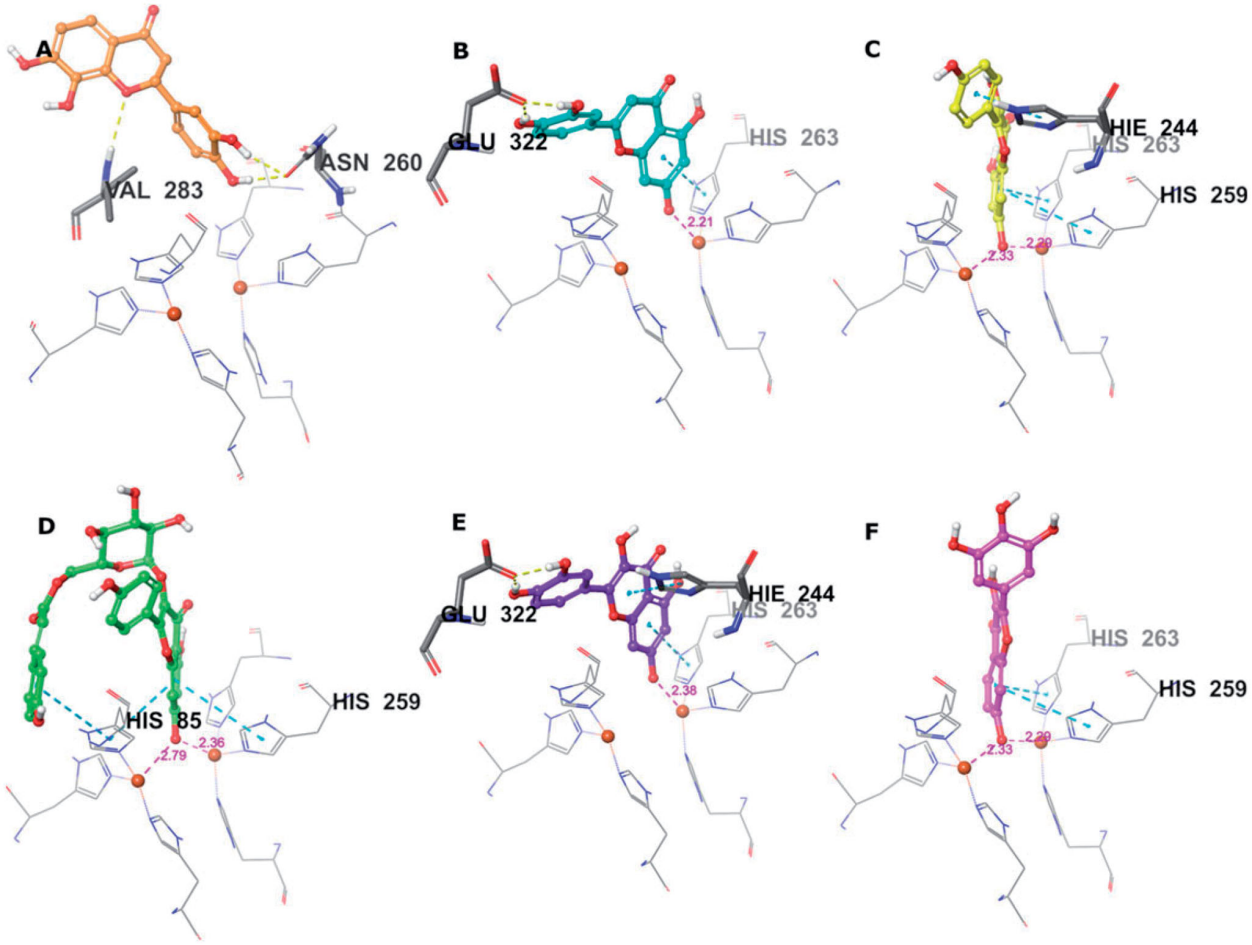
Predicted binding mode of compounds **4** (A), **17** (B), **26** (C), **32** (D), **35** (E), and **41** (F) in the tyrosinase active site. Compounds are represented as colour stick balls, interacting tyrosinase residues as grey sticks, Cu^2+^ as orange spheres, their histidine ligands as wireframes, and binding interactions as colour dashed lines. The distances between Cu^2+^ and interacting atoms are shown in Å.

Isookanin’s (**4**) binding mode lacked interactions with Cu^2+^ and its ligands (Figure 4(A)), which resulted in a moderate docking score. This result was most likely due to the lack of aromaticity (i.e., a double bond between C2 and C3) of its flavonoid core, which, in the case of other compounds, was able to reach deeper into the copper zone and make π-stacks with the key histidine residues. The lack of aromaticity in isookanin’s flavonoid core also cancelled out the compound’s planarity and led to bending, which may have made it sterically difficult to approach the copper ions.

**Figure 5.**
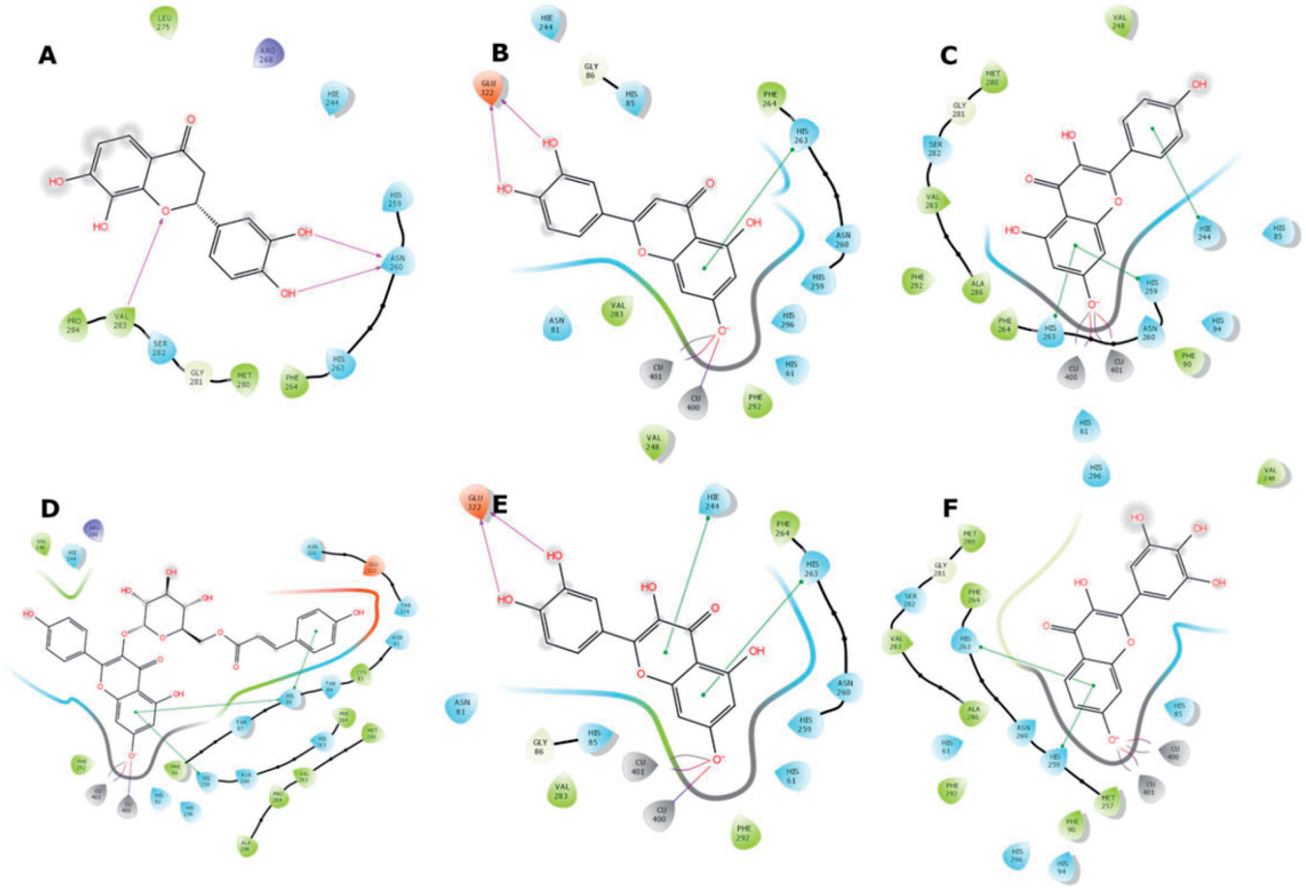
Binding interactions of compounds **4** (A), **17** (B), **26** (C), **32** (D), **35** (E), and **41** (F) with the tyrosinase active site residues. Binding interactions are shown as coloured lines, and compound moieties exposed to solvent are highlighted with grey shades.

Conversely, compounds **17**, **26**, **32**, **35**, and **41** were found to be suitable chelators of Cu^2+^ due to the common flavonoid core and the ionisable OH substituent at the 7th position (Figure 4(B–F)). The ionised hydroxyl was effective even for double Cu^2+^ engagement in the case of **26**, **32** and **41**, which were the best scoring compounds (see Table 3). Aromaticity of the flavonoid core enabled π stacking with the histidine residues serving as copper ligands, as well as His244. The hydroxyl groups on ring B of **17** and **35** formed H bonds with Glu322. The hydroxyphenyl attached to the glucopyranosyl moiety of **32** made additional π stacking with His85. Ring B of **17**, **26**, **35**, and **41** was observed to face active site entry and disposed to solvent molecules (Figure 5). With three hydroxyls on ring B, **41** was the most advantageous compound in this situation, which contributed to its docking score.

These results highlight the importance of the flavonoid core with a free hydroxyl at C-7 as a key component for copper chelation and for interactions with the key residues of the tyrosinase active site. The exceptional binding mode of **4** in the catalytic site with respect to its activity can be explained by more effective binding to a possible allosteric site.

### 3.4. Statistical modelling approach

To create a statistical model, 26 attributes (Table 4) that were consistent with all tested flavonoid compounds and their variables (IC_50_ values) were identified.

**Table 4.** Attributes definitions used to for the statistical analysis.

Number	Definition
A1	presence or absence of double bond between C2 and C3
A2	OMe substitution at C2’
A3	OH substitution at C3’
A4	OMe substitution at C3’
A5	OH substitution at C4’
A6	OMe substitution at C4’
A7	OAc substitution at C4’
A8	OH substitution at C5’
A9	OMe substitution at C6’
A10	OH substitution at C3
A11	OMe substitution at C3
A12	*O*-glc/*O*-gluc/*O*-rut/6-deoxy-*α*-L-man substitution at C3
A13	*O*-(6′'-*p*-Co)-glc substitution at C3
A14	OH substitution at C5
A15	OMe substitution at C5
A16	glc substitution at C6
A17	OH substitution at C7
A18	OMe substitution at C7
A19	*O*-rut/*O*-glc substitution at C7
A20	*O*-xyl-glc substitution at C7
A21	OH substitution at C8
A22	OMe substitution at C8
A23	glc substitution at C8
A24	3-Me-2-Bu substitution at C8
A25	glc-*O*-glc/xyl-*O*-glc substitution at C8
A26	(4′''acetoxy-xyl)-*O*-glc substitution at C8

Abbreviations: Me: methyl; B: butenyl; Ac: acetyl; glc: glucose; gluc: glucuronide; *p*-Co: *para*-coumaroyl; man: mannose; rut: rutinose; xyl: xylose.

Table 5 shows the multivariate linear regression model for the ln IC_50_. Statistical analysis explained more than 50% of the variability of the IC_50_ variable (adjusted R^2^=50.88%) with *p*= 0.0002. All 11 independent variables in this approach yield *p*< 0.2, while five of them (A4, A10, A18, A21 and A22) have a statistically significant influence on the dependent variable (IC_50_).

**Table 5.** The multivariate linear regression model for the ln IC_50_.

Attribute	Coefficient	e^Coefficient^	*p* Value	[95% Conf. Interval]	
A4	1.423116	4.15003	<0.001	0.7909708	2.055261
A5	0.3649103	1.44038	0.126	−0.1077861	0.8376067
A6	0.4055184	1.50008	0.148	−0.1517169	0.9627536
A10	−1.041826	0.35281	<0.001	−1.521979	−0.5616735
A13	−0.8969235	0.40782	0.088	−1.935625	0.1417784
A14	−0.4376985	0.64552	0.167	−1.068768	0.1933707
A15	−1.317022	0.26793	0.063	−2.71015	0.0761047
A18	0.9049781	2.47188	0.002	0.3562236	1.453733
A21	−1.630273	0.19588	0.008	−2.805552	−0.4549943
A22	1.452888	4.27544	0.012	0.3464813	2.559294
A25	0.5533549	1.73908	0.056	−0.0138585	1.120568

Adjusted R^2^=0.5088.

Based on these results, the presence of methoxy substitution at C3'(A4) increases the mean IC_50_ value by approximately 4.15 times, and increasing this value (by approximately 2.47 times) relates to methoxy substitution at C7 (A18). Methylation at C8 (A22) caused an increase in the IC_50_ value, as shown by lnIC_50_ (1.452888), which indicates that, in this case, the mean IC_50_ increases by approximately 4.28 times. Conversely, the presence of hydroxy substitution at C3 (A10) and hydroxy substitution at C8 (A21) reduced the mean IC_50_ by approximately 2.83 and 5.11 times, respectively. Overall, statistical modelling suggests that flavonoids with higher antityrosinase activity possess an OH group at C3 and at C8, while methylation of the hydroxyl group at carbon 3 and at carbon 8 notably reduces the inhibitory activity of the molecules.

## 4. Conclusions

Results from the literature that describe the effect of flavonoids on mushroom tyrosinase activity are dispersed and sometimes contradictory. This dissidence may relate to various experimental conditions (e.g., concentration of enzyme or substrate L-DOPA, wavelength), which led to different ranges of reported inhibitory activity. Furthermore, generally few flavonoids were included in such investigations, which yields restricted structure-activity relationships information. The statistical model developed in this study provides accurate SAR data in a comprehensive format. Many flavonoids were tested under the same conditions to obtain values that are as close to those in the real world as possible. In summary, a comparison of IC_50_ values, kinetic reactions, molecular docking scores and statistical data allowed us to identify the characteristics of flavonoid structures that facilitate tyrosinase inhibition: the presence of hydroxyls at C3 and C7, *O*- and *C*-glycosylation, methylation and acetylation of OH groups. As in many other cases reported in the literature^34^, the natural products represent a gold mine for interesting biological activities which can be translated to potential biomedical applications.

## References

[1] Jakimiuk K, Wink M, Tomczyk M. Flavonoids of the Caryophyllaceae. Phytochem Rev 2021;20:1–40.

[2] García-Sosa AR, Maran U. Improving the use of ranking in virtual screening against HIV-1 integrase with triangular numbers and including ligand profiling with antitargets. J Chem Inf Model 2014;54:3172–85. 25303089 10.1021/ci500300u

[3] Glisic S, Sencanski M, Perovic V, et al. Arginase flavonoid anti-leishmanial *in silico* inhibitors flagged against anti-targets. Molecules 2016;21:589–14. 10.3390/molecules21050589 PMC6274217 27164067

[4] Flurkey WH, Inlow JK. Proteolytic processing of polyphenol oxidase from plants and fungi. J Inorg Biochem 2008;102:2160–70. 18829115 10.1016/j.jinorgbio.2008.08.007

[5] Ismaya WT, Rozeboom HJ, Weijn A, et al. Crystal structure of *Agaricus bisporus* mushroom tyrosinase: identity of the tetramer subunits and interaction with tropolone. Biochem 2011;50:5477–86. 21598903 10.1021/bi200395t

[6] Klabunde T, Eicken C, Sacchettini JC, Krebs B. Crystal structure of a plant catechol oxidase containing a dicopper center. Nat Struct Mo. Biol 1998;5:1084–90. 10.1038/4193 9846879

[7] Zolghadri S, Bahrami A, Hassan Khan MT, et al. A comprehensive review on tyrosinase inhibitors. J Enzyme Inhib Med Chem 2019;34:279–309. 30734608 10.1080/14756366.2018.1545767 PMC6327992

[8] Hazes B, Magnus K, Bonaventura C, et al. Crystal structure of deoxygenated *Limulus polyphemus* subunit II hemocyanin at 2.18 A resolution: clues for a mechanism for allosteric regulation. Protein Sci 1993;2:597–619. 8518732 10.1002/pro.5560020411 PMC2142367

[9] Ferro S, Deri B, Germanò MP, et al. Targeting tyrosinase: development and structural insights of novel inhibitors bearing arylpiperidine and arylpiperazine fragments. J Med Chem 2018;61:3908–17. 29634898 10.1021/acs.jmedchem.7b01745

[10] Lai X, Wichers HJ, Soler-Lopez M, Dijkstra BW. Structure and function of human tyrosinase and tyrosinase-related proteins. Chem–An Eur J 2018;24:47–55. 10.1002/chem.201704410 29052256

[11] Şöhretoğlu D, Sari S, Barut B, Özel A. Tyrosinase inhibition by some flavonoids: inhibitory activity, mechanism by *in vitro* and *in silico* studies. Bioorg Chem 2018;81:168–74. 30130649 10.1016/j.bioorg.2018.08.020

[12] Isidorov V, Szoka L, Nazaruk J. Cytotoxicity of white birch bud extracts: perspectives for therapy of tumours. PLoS One 2018;13:e0201949–10. 30106978 10.1371/journal.pone.0201949 PMC6091957

[13] Nazaruk J, Jakoniuk P. Flavonoid composition and antimicrobial activity of *Cirsium rivulare* (Jacq.) all flowers. J Ethnopharmacol 2005;102:208–12. 16061337 10.1016/j.jep.2005.06.012

[14] Tomczyk M, Gudej J. Quercetin and kaempferol glycosides from *Ficaria verna* flowers and their structure studied by 2D NMR spectroscopy. Pol J Chem 2002;76:1601–5.

[15] Strawa J, Wajs-Bonikowska A, Jakimiuk K, et al. Phytochemical examination of woolly burdock *Arctium tomentosum* leaves and flower heads. Chem Nat Compd 2020;56:345–7.

[16] Juszczak AM, Czarnomysy R, Strawa JW, et al. *In vitro* anticancer potential of *Jasione montana* and its main components against human amelanotic melanoma cells. Int J Mol Sci 2021;22:1–25. 10.3390/ijms22073345 PMC8036727 33805898

[17] Strawa J, Galanty A, Jakimiuk K, Grabowska K, Podolak I, Tomczyk M. Cytotoxic effect on human melanoma cell lines and tyrosinase inhibition of *Hottonia palustris*. Poster session presented at: 69th International Congress and Annual Meeting of the Society for Medicinal Plant and Natural Product Research; 5-9 September 2021; Bonn, Germany; Virtual Conference.

[18] Jakimiuk K, Strawa JW, Granica S, Tomczyk M. New flavone C-glycosides from *Scleranthus perennis* and their anti-collagenase activity. Molecules 2021;26:5631–10. 34577102 10.3390/molecules26185631 PMC8468783

[19] Tomczyk M. Secondary metabolites from *Potentilla recta* L. and *Drymocallis rupestris* (L.) Soják (syn. *Potentilla rupestris* L.) (Rosaceae). Biochem Syst Ecol 2011;39:893–6.

[20] Gudej J, Tomczyk M. Polyphenolic compounds from flowers of *Ficaria verna* Huds. Acta Pol Pharm 1999;56:475–6.

[21] Ciganović P, Jakimiuk K, Tomczyk M, Zovko-Končić M. Glycerolic licorice extracts as active cosmeceutical ingredients: extraction optimization, chemical characterization, and biological activity. Antioxidants 2019;8:445–14. 10.3390/antiox8100445 PMC6826613 31581512

[22] Yoshino M, Murakami K. A graphical method for determining inhibition constants. J Enzyme Inhib Med Chem 2009;24:1288–90. 19912063 10.3109/14756360902829766

[23] Butt ARS, Abbasi MA, Rehman AA, et al. Synthesis and structure-activity relationship of tyrosinase inhibiting novel bi-heterocyclic acetamides: mechanistic insights through enzyme inhibition, kinetics and computational studies. Bioorg Chem 2019;86:459–72. 30772647 10.1016/j.bioorg.2019.01.036

[24] Lu C, Wu C, Ghoreishi D, et al. OPLS4: improving force field accuracy on challenging regimes of chemical space. J Chem Theory Comput 2021;17:4291–300. 34096718 10.1021/acs.jctc.1c00302

[25] Berman HM, Westbrook J, Feng Z, et al. The protein data bank. Nucleic Acids Res 2000;28:3908–17. 10.1093/nar/28.1.235 PMC102472 10592235

[26] Sastry GM, Adzhigirey M, Day T, et al. Protein and ligand preparation: parameters, protocols, and influence on virtual screening enrichments. J Comput Aided Mol Des 2013;27:221–34. 23579614 10.1007/s10822-013-9644-8

[27] Friesner RA, Murphy RB, Repasky MP, et al. Extra precision glide: docking and scoring incorporating a model of hydrophobic enclosure for protein-ligand complexes. J Med Chem 2006;49:6177–96. 17034125 10.1021/jm051256o

[28] Jacob V, Hagai T, Soliman K. Structure-activity relationships of flavonoids. Curr Org Chem 2011;15:2641–57.

[29] Cos P, Ying L, Calomme M, et al. Structure-activity relationship and classification of flavonoids as inhibitors of xanthine oxidase and superoxide scavengers. J Nat Prod 1998;61:71–6. 9461655 10.1021/np970237h

[30] Jakimiuk K, Gesek J, Atanasov AG, Tomczyk M. Flavonoids as inhibitors of human neutrophil elastase. J Enzyme Inhib Med Chem 2021;36:1016–28. 33980119 10.1080/14756366.2021.1927006 PMC8128182

[31] Zhang L, Zhao X, Tao G-J, et al. Investigating the inhibitory activity and mechanism differences between norartocarpetin and luteolin for tyrosinase: a combinatory kinetic study and computational simulation analysis. Food Chem 2017;223:40–8. 28069121 10.1016/j.foodchem.2016.12.017

[32] Fan M, Zhang G, Hu X, et al. Quercetin as a tyrosinase inhibitor: inhibitory activity, conformational change and mechanism. Food Res Int 2017;100:226–33. 28873682 10.1016/j.foodres.2017.07.010

[33] Lu Y, Chen J, Wei D, et al. Tyrosinase inhibitory effect and inhibitory mechanism of tiliroside from raspberry. J Enzyme Inhib Med Chem 2009;24:1154–60. 19772488 10.1080/14756360802694252

[34] Atanasov AG, Zotchev SB, Dirsch VM, Supuran CT. Natural products in drug discovery: advances and opportunities. Nat Rev Drug Discov 2021;20:200–16. 33510482 10.1038/s41573-020-00114-z PMC7841765

